# Development and Validation of an In-House Library for Filamentous Fungi Identification by MALDI-TOF MS in a Clinical Laboratory in Medellin (Colombia)

**DOI:** 10.3390/microorganisms8091362

**Published:** 2020-09-06

**Authors:** Juan C. Gómez-Velásquez, Natalia Loaiza-Díaz, Gilma Norela Hernández, Nelson Lima, Ana C. Mesa-Arango

**Affiliations:** 1 Laboratorio Clínico Grupo Synlab, Calle 19 A, Medellín 050021, Colombia; jugovela@gmail.com (J.C.G.-V.); natalia.loaiza@synlab.co (N.L.-D.); 2 Grupo Académico de Epidemiología Clínica, Facultad de Medicina, Universidad de Antioquia, Carrera 51 D, Medellín 050010, Colombia; gilma.hernandez@udea.edu.co; 3 CEB-Centre of Biological Engineering, Micoteca da Universidade do Minho (MUM), University of Minho, Campus de Gualtar, 4710-057 Braga, Portugal; 4 Grupo de Investigación Dermatológica, Instituto de Investigaciones Médicas, Facultad de Medicina, Universidad de Antioquia, Avenida Juan del Corral, Medellín 050010, Colombia

**Keywords:** in-house library, filamentous fungi, MALDI-TOF MS, clinical isolates

## Abstract

Identification of filamentous fungi by conventional phenotypic methods are time-consuming, and a correct identification at the species level is prone to errors. Therefore, a more accurate and faster time-to-results, and cost-effective technique, is required, such as the Matrix-Assisted Laser Desorption/Ionization Time-of-Flight Mass Spectrometry (MALDI-TOF MS). In this study, we describe the development of an in-house spectra library for the identification of filamentous fungi frequently isolated from patients with infections. An in-house spectra library was constructed using 14 reference strains grown in solid medium. Clinical isolates were identified either by the in-house spectra library or the Biotyper commercial library from Bruker Daltonics. Fungal identification was carried following the Biotyper’s established scores: ≤1.699: not reliably identified (NRI); 1.700–1.999: genus-level; ≥2.000: species-level. Clinical isolates were identified, with the in-house library, at species- and genus-level at 88.70% (55) and 3.22% (2), respectively. While 4.80% (3) was NRI and 3.22% (2) was discrepant concerning sequencing. On the contrary, identification up to species and genus-level with the commercial library was 44.44% (16) and 22.22% (8), respectively. NRI and the discrepancy was 30.55% (11) and 2.77% (1), respectively. For the reaming 26 isolates, 16 from *Neoscytalidium dimidiatum* and 10 from *Sporothrix* spp., respectively, the absence of spectrum and the specific spectra within the *Sporothrix* complex in the commercial library resulted in the inability to obtain an identification. In conclusion, the current results advocate the importance that each clinical microbiological laboratory needs to develop an ad hoc library associated with the MALDI-TOF MS fungal identification to overcome the limitations of the available commercial libraries.

## 1. Introduction

Infections by filamentous fungi species have become a health problem due to the high mortality and morbidity that they cause [1,2]. Rapid and accurate identification of these organisms is critical in terms of epidemiology and an adequate choice of therapy [3,4]. The conventional identification of fungi in the diagnostic mycological laboratories in developing countries is based mainly in isolated in vitro of the etiological agent from biological samples, and subsequent identification based on conventional phenotypic (macro- and micro-morphologies) traits [5,6,7]. Nevertheless, identification based on these methods has limitations, resulting in incomplete or erroneous identifications, mainly when very close related or cryptic species are involved [6,8,9].

Fungal infections by filamentous fungi, mainly of the genera *Aspergillus*, *Fusarium*, *Mucor,* or *Scedosporium*, are of low frequency but of high mortality [10] while dermatophytosis are the most frequent infections worldwide caused by keratinolytic filamentous fungi, mainly *Epidermophyton floccosum* or species of *Trichophyton* and *Microsporum* [11]. The identification of these fungi by conventional methods can result in misidentification due to the similarity between phylogenetically closely species, mainly within the complexes *Trichophyton mentagrophytes* and *T. rubrum* [12]; hence, the need for more robust techniques for their identification. The same occurs with the etiological agents of sporotrichosis, an implantation mycosis of interest in tropical countries, which can be caused by any of the four cryptic species that belong to the *Sporothrix schenckii* complex, among which there is a difference in antifungal susceptibility, virulence, and geographical distribution [13].

Although molecular techniques, such as sequencing of one or several DNA barcodes, are currently available to identify different fungal species [14,15], the use of this technique in routine diagnostic mycological laboratories in countries such as Colombia, is not yet an ordinary option. Currently, Matrix-Assisted Laser Desorption/Ionization Time-Of-Flight Mass Spectrometry (MALDI-TOF MS) is a cost-effective alternative for bacteria, mycobacteria, and yeast identification [16,17]. For filamentous fungi, since the first reports published in 2000 by Welham et al. [18] and Li et al. [19], where spores were analysed, many other subsequent contributions discussed developments for their identification by MALDI-TOF MS [20,21,22,23,24,25,26]. Subsequently, Seyfarth et al. [27] and Erhard et al. [28] published the first protocols for identification by MALDI-TOF MS of *Fusarium proliferatum* and dermatophytes, respectively. Santos et al. [24] recently review the impact of MALDI-TOF MS in clinical mycology laboratories pointing out the main progresses and barriers in fungal identification. These authors elected as limitations the commercial libraries with limited coverage of representative fungal taxa and the requirement to standardise procedures. MALDI-TOF MS uses for the identification of dimorphic or filamentous fungi is still a challenge since there are marked structural differences between genera or species, involving diverse procedures, or because of the lack of spectra in commercial libraries to identify endemic or frequent fungi causing infection in tropical regions such as is Colombia.

This study aims to describe the ground rules for development and validation of an in-house spectra library for the identification of filamentous fungi frequently isolated from patients with infections.

## 2. Materials and Methods

### 2.1. Reference Strains and Clinical Isolates

Fourteen reference strains from different microbial culture collections were used for the development of in-house spectra library (Table 1).

Afterwards, the validation of the developed in-house spectra library was carried out with 25 isolates representing 14 different species from the genus *Aspergillus*, previously identified by sequencing of the fragment the β-tubulin or calmodulin gene, *Microsporum*, *Nannizzia*, *Neoscytalidium,* and *Trichophyton*, identified by sequencing of the ITS1 + 5.8S + ITS2 nuclear rDNA region, *Sporothrix* identified with both β-tubulin and ITS1 + 5.8S + ITS2 DNA barcodes, and *Fusarium* identified by sequencing the translation elongation factor 1-α (TEF) gene region.

Finally, with the in-house library developed and validated and with the commercial library MALDI Biotyper (Bruker Daltonics, Bremen, Germany), 62 clinical isolates, previously identified by conventional phenotypic methods and by gene sequencing, were used to test the robustness of both libraries on identification: *N. dimidiatum* (16), *Microsporum canis* (11), *Trichophyton interdigitale* (11), *T. rubrum* (14), *Sporothrix globosa* (5), and *S. schenckii* s.s. (5). These isolates originate from clinical samples of the skin, nail, and hair sent for analysis by general practitioners or dermatologists to the microbiological laboratory. Therefore, they are essentially limited to dermatophytes fungi.

### 2.2. Molecular Identification

#### 2.2.1. Preparation of Mycelium for the Extraction of DNA

Fungi were grown at 28 °C during 5 days in potato dextrose agar (PDA; Oxoid, Basingstoke, Hampshire, England). Then, a 1.5-mL microtube (KIMA, Piove di Sacco, Padova, Italy) containing 1 mL of yeast malt broth (YMB; yeast extract, 3 g/L; malt extract, 3 g/L; peptone 5 g/L; glucose 10 g/L) was used to inoculate with approximately 0.2 cm^2^ of the biomass from the fungal colony. The microtubes were incubated for 10 days in constant agitation at room temperature. Afterwards, they were centrifuged at 16,089× *g* during 10 min, the supernatant was discarded, and 1 mL of sterile distilled water was added to remove residues from the YMB. They were centrifuged again and the supernatant poured-off. Then, the pellet was dried and kept at –20 °C, until it was used for DNA extraction.

#### 2.2.2. DNA Extraction

The protocol developed by Rodrigues et al. [29], with minor modifications for fungal DNA extraction, was performed as described in detail by Flórez-Munõz et al. [30] elsewhere.

#### 2.2.3. PCR Amplification and DNA Sequencing

PCR amplification of the ITS1 + 5.8S + ITS2 rDNA, β-tubulin, or TEF regions were performed with 25 μL of NZYTaq II 2x Green Master Mix (NZTtech, Lisbon, Portugal), 1 μL of each primer at 10 mM, respectively, ITS1/ITS4 [31], Bt2-F/Bt2-R [32], or EF1-728F/EF1-986R [33], 50 ng of template DNA in a 50 μL final reaction volume completed with sterile ultra-pure water. The same cycling conditions were used for the primer pairs ITS and Bt2 and were as follows: a denaturation step at 94 °C for 3 min; 35 cycles of the annealing step: 1 min at 94 °C, 1 min at 55 °C, and 1 min at 72 °C; and a final elongation step of 5 min at 72 °C. For the primer pair EF1, the PCR conditions were as follows: 95 °C for 5 min as the initial step followed by 35 cycles of denaturation at 95 °C for 15 s, annealing at 55 °C for 20 s, and a final extension at 72 °C for 60 s. PCR amplifications were conducted in a GeneAmp PCR System 9700 thermocycler (Applied Biosystems, Foster City, CA, USA).

To analyse the PCR products, electrophoresis with a 1% agarose gel was performed, and the amplicons purified using the NZYgelpure kit from NZYtech were sent for Sanger sequencing to Stab Vida Lda (Caparica, Portugal).

#### 2.2.4. DNA Sequence Processing and Molecular Identification

DNA sequences were analysed using the FinchTV program, version 1.4.0 (Geospiza, Akron, OH, USA), poor-quality end regions were removed and, finally, the fungal identifications obtained comparing the sequences against sequences available on GenBank from the National Center for Biotechnology Information (NCBI, Bethesda, MD, USA).

### 2.3. Protein Extraction and Development In-House Spectra Library

For the development of in-house spectra library, proteins were obtained according to the methodology proposed by Packeu et al. [34], with minor modifications. Four replicate solid medium cultures, of each strain indicated in Table 1, were grown on Sabouraud gentamicin chloramphenicol 2 (SGC2, ref. 43659, Biomerieux^®^, Paris, France). Before protein extraction, and to optimize the accuracy, the minimum growth time of each species at 28 ± 2 °C where the colonies did not present observational pigmentation and conidiation was standardized. On the optimal time, the young mycelium was removed gently near the edge of the colony with a wooden toothpick and suspended in a mixture of 900 μL of ethanol and 300 μL distilled water. The mixture was homogenized and centrifuged for 5 min at 16,089 × *g*. The supernatant was carefully removed and to the precipitate air- dried. Afterwards, 50 μL of formic acid (70%) was added to the precipitated, homogenized and left to stand for 15 min. Then, 50 µL of acetonitrile was added and left to stand for 15 min. This solution was centrifuged for 2 min at 16,089 × *g*. One µL of the obtained supernatant was taken and it was dropped in wheel positions on the steel target plate (Bruker Daltonics, Bremen, Germany). For each out of four replicates, this procedure was repeated twice. When the samples were dried, 1 µL of α-cyano-4-hydroxycinnamic acid (Bruker Daltonics, Bremen, Germany) was added. The spectra of each of the eight-well positions (technical replicates) were obtained using the FlexControl 3.3 tool (Bruker Daltonics, Bremen, Germany). Therefore, resulting in four reference spectra for each strain, each one comprised of at least 20 to 24 replica spectra, according to the manufacturer’s recommendations (Bruker Daltonics, Bremen, Germany).

### 2.4. Validation of In-House Library and Identification of Clinical Isolates

Spectra were validated with 25 strains covering 14 different species. The protein extraction of all fungi was performed as described above, and four samples of each fungus were analysed using MALDI Biotyper RTC software 3.1 version (Bruker Daltonics, Bremen, Germany). Identification was carried out according to manufacturer’s established scores: ≤1.699: not reliably identified (NRI); 1.700–1.999: genus-level identification; ≥2.000: species-level identification. It was required that at least three of the four samples analysed obtained a score that would allow them to be placed in the same category. Moreover, parallel identification with the MALDI Biotyper commercial library was carried out. Once the spectra were validated, 62 clinical isolates were analysed. From each isolated, four spectra were compared with in-house spectra library and with commercial library filamentous fungi, using MALDI Biotyper software 3.1 version (Bruker Daltonics, Bremen, Germany).

### 2.5. Statistical Analysis

The agreement between the identification of the strains or clinical isolate used for the validation, by in-house spectra library and by the commercial filamentous fungi library (Bruker Daltonics, Bremen, Germany) was determined by calculating the kappa coefficient of Cohen (*κ*) with the SPSS statistical package version 25. In addition, the agreement between the identification of clinical isolates by the classical method, gene sequencing or MALDI-TOF MS was analysed. The interpretation of (κ) value was performed according to the categories defined by Landis and Koch [35].

## 3. Results

The minimum growth time of the different fungi was that in which enough protein was obtained to achieve quality spectra. This time varied between 36 h to 96 h according to genus or species as shown in Table 2.

From the 14 species (Table 1) distributed by seven genera, including *Aspergillus* (6 species), *Fusarium*, *Microsporum*, *Nannizzia*, *Neoscytalidium*, *Sporothrix* (2 species), and *Trichophyton* (2 species), fifty-six reference spectra were successfully created.

**Figure 1:**
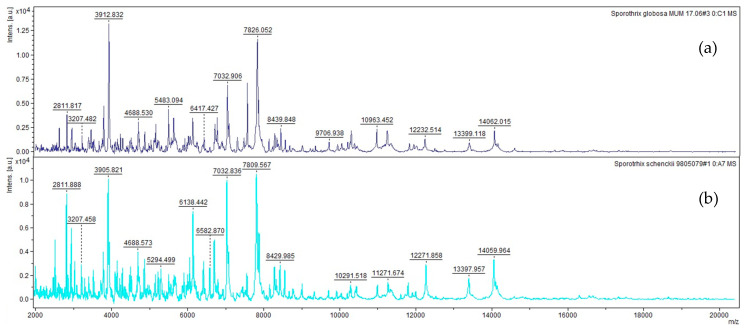
References spectra between 2 and 20 kDa of (**a**) *S. globose* and (**b**) *S. schenckii* s.s.

Within the *Sporothrix* complex, the reference spectra of *S. globosa*, and *S. schenckii* s.s. are shown as examples in Figure 1. Each image corresponds to a spectrum out of the four that constitute the fungal species reference spectra. Each spectrum shows clear differences in the proteins profiles (fingerprinting) obtained between 2 and 20 kDa allowing a good delimitation between these two close related species.

Afterwards, and for validation of the in-house library, spectra from 25 strains were generated as indicated in Table 3. Scores for validation of in-house spectra library against the identification by the commercial library for filamentous fungi (Bruker, Daltonics, Germany) show that the identifications by the in-house spectra library were much more reproducible (24 out of 25 with 4/4) than in the commercial library (2 out of 25 with 4/4 and 2 with 3/4). Only *N. gypsea* strain was not identified using the in-house library, as it is not in the commercial library.

Finally, for full operation with MALDI-TOF MS in-house library, 55 out of 62 (88.70%) clinical isolates were identified at species level, 2 isolates (3.22%) at genus level identification and 3 isolates (4.80%) with NRI. The last 3.22% (2 isolates) were discrepant with regard to molecular identification. By contrast, with the commercial library for the same set of 62 clinical isolates, 16 isolates of *N. dimidiatum*, 5 *S. globosa,* and 5 *S. schenckii* s.s. were not identified because any spectrum from the *N. dimidiatum* was not available in the library for comparison, and the *S. schenckii* commercial spectrum was not able to discriminate species within the *Sporothrix* complex, giving NRI low score (≤1.699) for all 10 isolates. For the 36 remain isolates, dermatophytes including *M. canis*, *T. interdigitale,* and *T. rubrum*, the identification up to species and genus was 44.44% (16) and 22.22% (8), respectively, NRI 30.55% (11), and discrepant 2.77% (1).

According to the categories used by Landis and Koch [29], the agreement between genus identification by classic phenotype and sequencing versus MALDI-TOF MS was almost perfect (*κ* = 0.990) while at the species-level was substantial perfect (*κ* = 0.595). In contrast, the agreement to the species-level identification by classic phenotype and sequencing versus MALDI-TOF MS with in-house spectra library, and with the commercial library was substantially perfect (*κ* = 0.728), and medium (*κ* = 0.218), respectively. Both libraries showed inconsistencies to identify *T. interdigitale*.

## 4. Discussion

Identification of filamentous fungi by conventional phenotypic (macroscopic and microscopic) traits must be supported by more accurate techniques. Although, the sequencing of one or more targets is more sensitive and specific than classical methods. Furthermore, this technique is not cost-effective in routine diagnostic mycological laboratories in developing countries [36,37,38].

MALDI-TOF MS has been recognized as an accurate, faster, time-to-results, as well as cost-effective, providing an excellent method for microbial identification. However, the application for filamentous fungi identification can be more complicated for different reasons, as follows: (i) to obtain quality protein extracts from mycelium with different structural characteristics; (ii) there are no reference spectra of some species in commercial libraries, mainly of endemic or frequent fungi isolates in tropical regions; (iii) it is difficult to achieve the manufacturer’s established scores with some species [36,39,40].

Previous studies regarding the identification of filamentous fungi by MALDI-TOF MS show differences in growth times of fungi, protein extraction methods, and construction of reference spectra methodologies [41,42,43,44]; thus, results vary and are difficult to compare. In this study, a solid medium (Sabouraud chloramphenicol; Biomerieux^®^, Paris, France) was used to obtain the fungal mass for protein extraction, unlike the liquid medium manufacturer’s recommended (Bruker Daltonics, Bremen, Germany). In addition, minimum times of fungal growth were standardized to avoid the appearance of pigments and conidia since those conditions can interfere with MALDI-TOF MS fungal identification [42,45]. The melanin synthesis by *Sporothrix* spp., *N. dimidiatum*, *A. niger,* and *A. tubingensis* was prevented using young mycelium. Even though melanin appearances in *Sporothrix* spp. was avoided with 36 h of growth, yet conidiation was not, but this latter condition did not prevent the creation of reference spectra and the correct identification to species level was done. Most significantly, we were able to correctly identified *Sporothrix* species in even lesser time (36 h) using the mycelial phase, unlike Oliveira et al. [46], who used the yeast phase, thus, delaying the identification by six days.

By comparison, the creation of the reference spectra and identification of clinical isolates of the dematiaceous fungus *N. dimidiatum*, an important agent of onychomycosis and dermatomycosis [30,47], was done with 72 h of growth while Alshawa et al. [48], used cultures of three weeks of growth. Decreasing time identifying clinically important fungi is crucial for early initiation of therapy, or to control sources of infection.

Protein extraction is an essential step for correct identification of filamentous fungi by MALDI-TOF MS. Although, some researchers have done it directly from in vitro cultures [40,49], our experience showed that protein extraction was necessary. We suggest that using the mycelium directly prevents a homogeneous distribution of the samples on the steel target plate on which the laser hits; consequently, identification results may be inaccurate.

Creating in-house libraries is necessary both for the inclusion of genera or species prevalent in each country region or for the identification of endemic fungi not included in commercial libraries. To date, there is no consensus on the criteria for the construction of reference spectra or identification of clinical isolates, such as: (i) choice of the number of strains for the construction of the reference spectra; (ii) definition of the number of spectra for the construction of the reference; (iii) definition of the minimum number of mass spectra that must form the reference and the frequencies of these; (iv) the number of samples to be analysed for each fungus to be identified; (v) definition of scores for identification to genus or species level. Consequently, making it difficult to compare results between different studies.

The conditions used in this research, for the creation of the in-house spectra library, were alike with those used by Cassagne et al. [39]. Identification of our clinical isolates set reached to the genus and species level of 3.22% (2) and 88.70% (55), respectively; while with the commercial library was 44.44% (17) and 22.22% (8). Different criteria than those used in this study, have been applied by other authors, e.g., reference spectra have been created with less than 20 spectra or with mass numbers and frequencies lower than 70% or 75.0%, respectively [39,40,41,43,50,51]. It is likely that the difference in the defined criteria for each researcher explains the variability in identification results. While with the current in-house spectra library, the 88.70% (55) of clinical isolates were identified to the species, with the created by Becker et al. [41] and Normand et al. [51], the identification was of 50.90% and 85.60%, respectively. In these cases, the frequency was not a relevant consideration in the reference spectra. We believe that the frequency is an important factor to consider since it providing an estimate of the homogeneity and reproducibility of the spectra that comprise reference spectra.

On the other hand, the high percentage of identification to the species, 85.6%, achieved by Becker et al. [41], maybe because the score accepted to define species was ≥1.700, whereas in this study it was ≥2.000. The scores to defined genus or species also vary widely in the literature [25,42,43,50], in some cases, they are defined by each investigator e.g., Zvezdanova et al. [40], and Normand et al. [50], chose scores between 1.600–1.799 and ≥1.800 for identification to genus or species level, respectively. In contrast, we used the scores recommended by the manufacturer. Other authors have suggested considered lower scores if there is an agreement with the gene sequencing [41,50].

Other aspects that vary among studies are the number of analysed samples for the identification. In some cases, the analysis is done by duplicate or quadruplicate [39,41]. However, given the variability that may exist between the results of the same strain, increasing the number of samples can decrease the error. We analysed the proteins from clinical isolate by quadruplicate. Likewise, the number of samples analysed for each isolate or strain, and the criteria selected to accept the identification vary among studies.

For some researcher, it is enough that two of four samples fall in the same range, while others the high value is chosen [40,50]. We accepted the proposal of Cassagne et al. [39], at least three of four results had to have a score in the range that enable identification to the genus, species, or those not reliably identified.

The commercial library is composed of several spectra from different strains of the same species, while our in-house spectra library was constructed from four replications of the same strain (biological replicates). However, the identification with the former library did not surpass the latter. This situation is illustrated with the identification of *A. niger* in the validation process, with the in-house spectra library the two strains were identified to the species with the commercial library only one (MM-132, Table 3) was identified to the species, even though it has 12 reference spectra constructed with different strains.

Nowadays the increasing new species causing infections in humans is evident [52,53]. Likewise, changes in fungal taxonomy with the consequent emergence of complex and sections of cryptic species [54,55]. These situations alert to the need to constantly expand libraries.

In this study, in-house spectra library was constructed to identified species of the complex *S. schenckii* because the commercial library has a reference spectrum for *S. schenckii* identification, but it is not clear whether it was constructed with strains of S. *schenckii* s.s. or any another species of the *S. schenckii* complex. It was important for us to have reference spectra for the identification of de main species of the *S. schenckii* complex because it is locally known that at least two species of the complex *S. schenckii* have been isolated from patients with sporotrichosis [56,57]. For this reason, *S. brasiliensis* and *S. luriei* are envisage to be incorporated the in-house library to complete this complex. In addition, other species within this genus involved in human’s infections are *S. mexicana* [58], *S. chilensis* [59] and *S. pallida* s.s [60]. The former one was yet incorporate in-house library and the other ones will be as soon as reference strains be make available.

The identification of dermatophytes by classical methods continues to be a challenge due to the morphological similarity among them, the lack of a clear definition of species within complexes and the taxonomic proximity [54,55]. An example is *T. interdigitale*, a species that is indistinguishable by micro- and macro-morphological characteristics from others of the *T. mentagrophytes* complex. In addition, various studies have focused on the identification by MALDI-TOF MS of dermatophytes isolated of clinical samples using different commercial platforms and pre-analytical methods, such as fungal growth time, culture media direct identification or from protein extracts [61,62,63,64,65]. Nevertheless, the identification at species-level with commercial libraries is low and variable between studies (14% and 62%) [63,65,66]. This has led to in-house developed libraries in order to increase coverage and improve accurate identification of this group [67].

In our experience with MALDI-TOF MS identification, both the reference spectra creation and validation of the *M. canis*, *T. interdigitale* and *T. rubrum* species were successful. However, not all clinical isolates of *T. interdigitale* were correctly identified by both libraries. This difficulty has been warned by the manufacturer and it has been reported in other studies [61,62]. Besides, with MALDI-TOF MS from other commercial brands, existing difficulties that affect the high identification accuracy of species within *T. mentagrophytes* complex and *T. tonsurans,* which are often cross-identified with *T. interdigitale* [68]. In addition, these authors, using a large set of dermatophytes (72 strains) from the American Type Culture Collection (ATCC) mycology collection (Manassas, Virginia, USA), found that the three phylogroups of *Arthroderma benhamiae* (known also by its anamorphic name *Trichophyton benhamiae*) were well distinguished by MALDI-TOF MS from one another with high identification accuracy [68]. As stated by Ollivier and Ranque [61], the major peaks in *T. tonsurans* mass spectra are also present in *T. interdigitale,* which means that only with expansion of the reference spectra library by using an in-house spectra library to incorporate inter- and intra-specific dermatophyte diversity it will be possible overcome this intrinsic limitation.

Aspergilli and fusaria were not submitted to robustness test since it is out of the microbiological laboratory scope preserve at long-term isolates and, ultimately, depends on the samples received to perform fungal identifications. However, the in-house library is prepared to analyse promptly several species within these two genera upon arriving of the samples and the validation process gave 100% of the success identification at species level for the strains used (Table 3).

## 5. Conclusions

The current results advocate the importance that each clinical microbiological laboratory needs to develop an ad hoc library associated with the MALDI-TOF MS fungal identification to overcome the limitations of the available commercial libraries. The strategy here reported shows that the molecular biology for fungal identification remains the gold-standard method and is crucial when a quality control and quality assurance are put in place to develop and validate an in-house library for filamentous fungi identification by MALDI-TOF MS in a clinical microbiological laboratory. Even with a well-defined MALDI-TOF MS protocol to the filamentous fungi identification to make more accurate the results obtained, the main constrain to achieve an unambiguous identification remain connect with the quality and extension of the library used. Only with an investment to develop and enlarge an in-house library, it is possible the clinical microbiological laboratories be a partner in the identification of etiological agents of fungal infections. For this, reference strains from well-recognised international microbial resources centres should be easily accessed, and the molecular biology identification approach should also be manageable to implement what, in this current article, is recommended. For instances, species from the genera *Mucor*, *Rhizopus,* or *Scedosporium* or even *Fusarium solani* need to be incorporated in this in-house library as soon as appropriated. In general, all of the legal framework to exchange strains needs to be complied with special attention to the provisions related to Nagoya Protocol on access and benefit sharing [69]. Since 1995, Colombia is a party of the Convention on Biological Diversity but, so far, is not yet a party of this Nagoya Protocol [70]. Last, but not least, in tropical developing countries with odd fungal phenotypical intraspecific variability, to pursue this partnership, qualified human resources able to investigate and to implement all strategies reported here is of paramount importance.

## Figures and Tables

**Table 1:** Reference strains used in the development of an in-house library. In brackets, the gene region used for the fungal identification and the access number in the GenBank.

Strain	Strain
*Aspergillus flavus* MUM 10.200 (ITS/HQ340101)	*Microsporum canis* MUM 09.17 (ITS/JX122187)
*Aspergillusfumigatus* MUM 16.03 (ITS/MT422118)	*Nannizzia gypsea* (formerly *Microsporum gypsum*) MUM 10.135 (ITS/JX101932)
*Aspergillus lentulus* CM-1290 (TUB/EU310839)	*Neoscytalidium dimidiatum* MUM 17.21 (ITS/MN371274)
*Aspergillus niger* MUM 92.13 (ITS/KU729033)	*Sporothrixglobosa* MUM 17.06 (ITS/KP017084)
*Aspergillus terreus* MUM 9409 (ITS/KF278453)	*Sporothrix schenckii* s.s. MUM 17.25 (ITS/MT422119)
*Aspergillus tubingensis* MM-141 (CAM/MT876622)	*Trichophyton interdigitale* MUM 09.21 (ITS/JX122255)
*Fusarium oxysporum* MUM 14.05 (TEF/MT536776)	*Trichophyton rubrum* MUM 09.12 (ITS/JQ663981)

CM: Collection of Mycelial Fungi, National Center for Microbiology Carlos III Health Institute (Madrid, Spain); MM: Collection of National Autonomous University of Mexico (Mexico, DF, Mexico); MUM: Micoteca da Universidade do Minho (Braga, Portugal); CAM: calmodulin gene region; ITS: Internal Transcribed Spacer rDNA gene region; TEF: translation elongation factor 1-α gene region; TUB: β-tubulin gene region; s.s.: sensu stricto.

**Table 2:** Minimum growth times of different fungal taxa for protein extraction and reference spectra construction.

Fungi	Growth Time (h)
*Sporothrix* spp.	36
*Aspergillus* spp., *F. oxysporum*, *N. gypsea* and *T. interdigitale*	48
*N. dimidiatum*	72
*M. canis* and *T. rubrum*	96

**Table 3:** Score values of the validation of in-house spectra library and the identification by the commercial library for 25 filamentous fungi covering 14 fungal species.

Fungi	Identification by MALDI-TOF					
	Library					
	In-House			Commercial		
	NRI ≤1.699	Genus 1.700–1.990	Species ≥2.000	NRI ≤1.699	Genus 1.700– 1.990	Species 2.000≥
*A. flavus* ATCC 204305 *A. flavus* Plab 01301266			4/4 4/4	1/4 2/4	3/4 2/4	
*A. fumigatus* ATCC 204305 *A. fumigatus* CBS 144.89			4/4 4/4		3/4	4/4 1/4
*A. lentulus* MM-7152 *A. lentulus* MM-7140			4/4 4/4	NA NA		
*A. niger* MM-132 *A. niger* MM-129			4/4 4/4	4/4 4/4	1/4	3/4
*A. terreus* CDC 315			4/4		1/4	3/4
*A. tubingensis* MM-141			4/4	NA		
*F. oxysporum* ATCC48112 *F. oxysporum* CNSG			4/4 4/4	4/4	4/4	
*M. canis* Plab 04080969 *M. canis* Plab 8101402			4/4 4/4	2/4	1/4	4/4 1/4
*N. gypsea* Plab 10020410	4/4			4/4		
*N. dimidiatum* Plab 6232194 *N dimidiatum* Plab 1120471			4/4 4/4	NA NA		
*S. schenckii* s.s. UDEA 15565 *S. schenckii* s.s. UDEA 7027			4/4 4/4	4/4 4/4		
*S. globosa* UDEA 0004 *S. globosa* UDEA 14879			4/4 4/4	NA NA		
*T. interdigitale* ATCC 24198 *T. interdigitale* Plab 9050951			4/4 4/4	1/4 2/4	1/4	2/4 2/4

*T. rubrum* ATCC 28188 *T. rubrum* Plab 8191487			4/4 4/4	4/4 1/4	2/4	1/4

ATCC: American Type Culture Collection (Manassas, VA, USA); CBS: Westerdijk Fungal Biodiversity Institute, Utrecht, The Netherlands; CNSG: Centro Nacional de Secuenciación Genómica, University of Antioquia (Medellin, Colombia); MM: Collection of National Autonomous University of Mexico (Mexico, DF, Mexico); UDEA: University of Antioquia (Medellin, Colombia); CDC: Center for Disease Control and Prevention (Atlanta, GA, USA); Plab: SYNLAB Clinical Laboratory (Medellin, Colombia); NA: Not available; MALDI-TOF: Matrix-Assisted Laser Desorption/Ionization Time-of-Flight; NRI: not reliably identified.
